# Oral Anticoagulation in Patients With Atrial High-Rate Episodes: Focus on Clinical Implications

**DOI:** 10.7759/cureus.46686

**Published:** 2023-10-08

**Authors:** Smaro Dimou, Vasiliki C Mystakidi, Sotirios Chiotis, Stylianos Daios, Charalambos Kalantzis, Nikias Milaras, Theodoros D Karamitsos, Haralambos Karvounis, Georgios Efthimiadis, Stylianos Paraskevaidis

**Affiliations:** 1 First Department of Cardiology, AHEPA Hospital, Aristotle University of Thessaloniki, Thessaloniki, GRC; 2 Department of Cardiology, 424 General Military Hospital, Thessaloniki, GRC; 3 Third University Department of Cardiology, National and Kapodistrian University of Athens, Athens, GRC; 4 First University Department of Cardiology, National and Kapodistrian University of Athens, Athens, GRC

**Keywords:** oral anticoagulation, bleeding, stroke, atrial fibrillation, atrial high-rate episodes

## Abstract

Background

Although previous studies showed that atrial high-rate episodes (AHREs) are associated with a higher risk of developing incident atrial fibrillation (AF) and thromboembolic events, their clinical significance is still unclear. The purpose of this study was to define whether there is any clinical impact on the occurrence of ischemic and hemorrhagic events in patients with AHREs and initiation of oral anticoagulation (OAC).

Methodology

Patients with AHREs who had received cardiac implantable electronic devices (CIEDs, i.e., dual-chamber pacemaker [PM] or implantable cardioverter defibrillator [ICD]) were included in the study. OAC initiation was decided by the assistant doctor. Patients who received OACs comprised the OAC group, while patients who were not referred for OAC initiation were included in the control group. The primary endpoint was the time to the event of the occurrence of thromboembolic events (thromboembolic event-free survival).

Results

A total of 154 individuals (77 in each group) were enrolled in the study, with a mean age of 72.5 years. The mean follow-up period for the OAC group was 19.1 months and for the control group, 18.9 months (*P =* 0.9). Thromboembolic events were noticed only in seven patients. Six of them were in the control group, and only one in the OAC group (*P =* 0.05). Major bleeding events were noticed in five patients, one of whom was in the control group and the rest in the OAC group (*P *= 0.17).

Conclusions

OAC therapy in patients with AHREs was not associated with a significant difference in the risk of thromboembolic and bleeding events. Baseline patient characteristics and AHRE duration may be useful to intensify the monitoring and management of patients with AHREs. Bleeding events may be indicators of cancer in patients with AHREs receiving OACs.

## Introduction

The expanding use of cardiac implantable electronic devices (CIEDs) and their exceptional ability to monitor atrial rhythm reinforce their utilization in recording atrial arrhythmias. Atrial high-rate episodes (AHREs) are defined as atrial tachyarrhythmia episodes with a rate over 190 beats per minute (bpm) and duration ≥6 minutes, detected by CIEDs [1,2]. As studies have shown, the incidence of AHREs in patients with CIEDs is approximately 20% [3,4] and is constantly increasing through the years parallel to the rapid increase of CIEDs.

The clinical significance of AHREs is still unclear although some studies showed that they are associated with a threefold higher risk of developing incident atrial fibrillation (AF) and a twofold increase in thromboembolic events [4-6]. Given the lack of robust data, the management of this type of arrhythmia is unclear. One recently published randomized controlled trial (RCT), the NOAH-AFNET 6 (Non-Vitamin K Antagonist Oral Anticoagulants in Patients With Atrial High-Rate Episodes) trial, demonstrated that anticoagulation with edoxaban did not significantly reduce the incidence of cardiovascular death, stroke, or systemic embolism as compared with placebo and led to higher rates of major bleeding in patients with AHREs [7]. One more ongoing RCT is expected to provide further evidence regarding the appropriate management of AHRE episodes of six-minute to 24-hour duration detected by CIEDs (NCT:01938248) [8].

The purpose of this study is to define if there is any clinical impact on the occurrence of ischemic and hemorrhagic events in patients with AHREs and initiation of oral anticoagulation (OAC).

## Materials and methods

Study population

This study is a prospective nonrandomized clinical trial. In brief, from January 1, 2016, to September 30, 2021, patients who had received CIEDs, such as dual-chamber PMs or implantable cardioverter defibrillators, and in whom AHREs were detected were invited to take part in the study. The initiation of OAC was decided by the assistant doctor. Patients who received OACs comprised the OAC group, while patients who were not referred for OAC initiation were included in the control group. Inclusion criteria were as follows: (1) permanent PM, ICD, or cardiac resynchronization therapy PMs or defibrillators (CRT-P/D) capable of recording AHREs at the baseline assessment, (2) documentation of at least one episode of AHRE, and (3) age ≥18 years. Patients who had a CHA2DS2-VASc ≤ 1, clinical AF documented by surface 12-lead electrocardiogram (ECG) or Holter monitoring lasting ≥30 seconds, or who were already in treatment with OAC for other reasons were excluded from the analysis.

Demographic data, cardiovascular risk factors, CHA2DS2-VASc and HAS-BLED scores, ECGs, 24-hour Holter monitoring, and medication were recorded for each patient. Hypertension and diabetes mellitus were defined by European guidelines. All patients were followed up for at least 12 months for thromboembolic events, major or minor bleeding events, and occurrence of AF.

All individuals provided informed consent. The study was approved by the Medical Research Ethics Committee of the AHEPA University Hospital, Aristotle University of Thessaloniki.

Definitions

AHREs were defined as atrial tachyarrhythmia episodes with a rate over 190 bpm and duration ≥6 minutes, detected by CIEDs [1]. Ischemic stroke was defined as a focal neurologic dysfunction of sudden onset that persisted for more than 24 hours, corresponded to a vascular territory, and was confirmed by computed tomography (CT), showing features consistent with focal brain infarction [9]. TIA was defined as a transient episode of neurologic dysfunction caused by focal brain, spinal cord, or retinal ischemia without acute infarction [10]. A new occurrence of AF was defined as AF lasting more than 30 seconds and documented by a standard 12-lead ECG or a new 24-hour Holter monitoring. The review and diagnosis of both AHREs and AF episodes were conducted by a physician.

Major bleeding was defined, per the criteria set by the International Society on Thrombosis and Hemostasis (ISTH), as overt bleeding associated with a hemoglobin drop of 2 g/dL or more, necessitating a transfusion of 2 or more units of blood, occurring in a critical site, or contributing to death [11].

Patient follow-up and outcome measures

Patients in both groups were followed up for at least 12 months with either clinic visits or telephone contacts. The primary endpoint was the time to the event of occurrence of thromboembolic events (thromboembolic event-free survival).

Statistical analysis

Continuous variables were presented as means and standard deviations (SD) or as median with interquartile range (IQR) in case of non-normally distributed data and compared using independent samples t-test or Mann Whitney U test for normally distributed and skewed variables respectively. The normality of the data distribution was assessed by the Shapiro-Wilk test. Categorical variables were presented as frequencies and percentages and compared using the Chi-square test or Fisher’s exact test. Kaplan-Meier curves with a log-rank test were used to determine the presence of statistically significant differences according to OAC status. The significance level was set to a two-tailed *P*-value < 0.05. Data analysis was performed using the IBM SPSS Statistics for Windows, Version 24.0 (IBM Corp., Armonk, NY) program.

## Results

A total of 154 individuals (77 in each group) were enrolled in the study (with a mean age of 72.5 years and a mean duration of AHREs 44.5 minutes). The clinical characteristics of the patients classified according to the examined groups are displayed in Table 1. In terms of left atrial (LA) diameter, no statistical significance was noticed between the two groups. The duration of AHREs in the two groups also had no statistically significant differences.

**Table 1:** Main characteristics of patients with thromboembolic event/major bleeding. OAC, oral anticoagulant

Serial number	OAC	Age (years)	Duration of an episode (minutes)	CHA2DS2-VASc score	Event	Diagnosis
1	Rivaroxaban	80	185	4	Thromboembolic	Stroke
2	-	76	700	4	Thromboembolic	Stroke
3	-	82	2,350	4	Thromboembolic	Stroke
4	-	84	1,200	4	Thromboembolic	Stroke
5	-	83	1,005	3	Thromboembolic	Stroke
6	-	88	2,200	5	Thromboembolic	Stroke
7	-	82	3,120	4	Thromboembolic	Stroke
8	Dabigatran	66	837	4	Bleeding	Colorectal cancer
9	Rivaroxaban	61	6.2	4	Bleeding	Colorectal cancer
10	Apixaban	81	21	5	Bleeding	Gastrointestinal bleeding
11	Rivaroxaban	79	303	4	Bleeding	Gastric cancer
12	-	88	122	5	Bleeding	Prostate cancer

**Figure 1:**
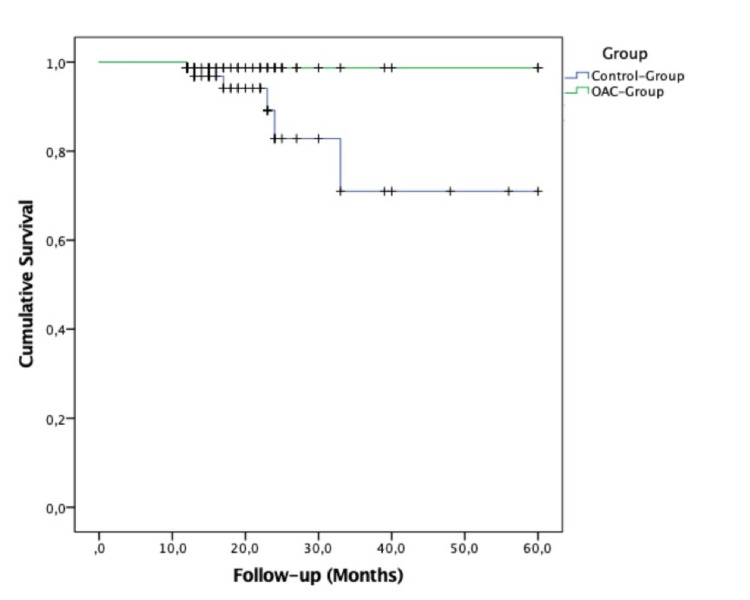
Kaplan-Meier curve for thromboembolic event-free survival according to OAC status (primary endpoint). OAC, oral anticoagulant

The mean follow-up period for the OAC group was 19.1 months, and for the control group, it was 18.9 months (*P* = 0.90). In that period, thromboembolic events were noticed only in seven patients of the examined groups, six of whom were in the control group and only one in the OAC group (*P* = 0.05). Regarding the secondary endpoints, major bleeding events were noticed in five patients in the total of examined patients, one of them in the control group and the rest in the OAC group (*P* = 0.17) (Table 1). Moreover, four of the five patients who experienced a bleeding event were diagnosed with cancer. The main characteristics of these patients are displayed in Table 2. Also, in total, 10 patients died during the follow-up period. In the OAC group, four patients had clinical AF in contrast to six patients in the control group (*P* = 0.75). In the log-rank analysis, patients had a thromboembolic event on the 59th day of follow-up in contrast to a control group who had sooner, on the 50th day (*P* = 0.06) (Figure 1).

**Table 2:** Study participants’ demographic, clinical, and laboratory characteristics. OAC, oral anticoagulant; IQR, interquartile range; ACEi, angiotensin-converting enzyme inhibitor; ARB, angiotensin receptor blocker; ARNI, angiotensin receptor/neprilysin inhibitor

Variables	OAC group (*N *= 77)	Control group (*N *= 77)	*P*-value
Age (years, IQR)	75 (69-79)	71 (61.5-82)	0.31
Male gender (%)	59.7	68.8	0.26
Dyslipidemia (%)	59.7	68.8	0.24
Hypertension (%)	89.6	72.7	0.01
Diabetes mellitus (%)	27.3	31.2	0.60
Coronary/peripheral artery disease (%)	27.3	49.4	0.01
Thyroid dysfunction (%)	10.4	20.8	0.08
ACEi/ARB/ARNI (%)	67.5	59.7	0.32
Diuretics (%)	45.5	45.5	1.00
Beta-blockers (%)	61	55.8	0.51
Calcium channel blockers (%)	37.7	19.5	0.01
Left atrial diameter (mm, IQR)	42 (38-45)	45 (38-48.5)	0.16
Left ventricular ejection fraction (%, IQR)	55 (45-55)	50 (35-60)	0.16
Duration of AHREs (minutes, IQR)	45 (10.5-256.5)	44 (9-197.5)	0.54
Events			
Thromboembolic event	1	6	0.05
Bleeding	4	1	0.17
Death	4	6	0.51
Atrial fibrillation	5	6	0.75

## Discussion

In this study, the group without an OAC treatment experienced higher rates of thromboembolic events and fewer rates of bleeding events than the OAC group; however, the statistical significance was not important (*P* = 0.05 and *P* = 0.17 for thromboembolic and bleeding events, respectively). An anticoagulant-related bleeding event was more likely to be an undiagnosed cancer. In the log-rank analysis, patients in the OAC group had a thromboembolic event on the 59th day of follow-up in contrast to the control group who had sooner, on the 50th day (*P* = 0.06).

Several studies have shown the association between AHREs and thromboembolic and bleeding events in patients with CEIDs and reported that patients with AHREs are more susceptible to developing clinical AF, heart failure (HF), and thromboembolic events than those without episodes [4,6,12-17]. However, in our study, we could not statistically prove that treatment with OACs in patients with AHREs reduces the risk for thromboembolic events, and we found no significant difference in the incidence of bleeding events between the studied groups. Also, during the follow-up, three of the four patients who had a bleeding event in the OAC group and one in the control group were diagnosed with different types of cancer [18]. Our study's observations regarding thromboembolic events are consistent with the largest RCT available, namely, the NOAH-AFNET 6 trial, where a statistically significant reduction in thromboembolic events was similarly not observed [7]. Interestingly, our study diverges in terms of bleeding events, where we did not observe a statistically significant increase, in contrast to the NOAH-AFNET 6 study. This difference may be attributed to the comparatively smaller sample size in our study and, subsequently, the limited number of recorded outcomes.

The most important question that is addressed in the decision of OAC initiation is the duration of the AHRE. Lu and Chen reported that patients with a longer AHRE duration had a higher risk of a major adverse cardiovascular event (MACE), especially acute coronary syndrome, when they also had a history of AF or myocardial infarction (MI), and the cutoff point was found in duration less or over 5 minutes [13]. They also noticed that patients with a history of MI may have a lower cutoff point for predicting MACE than those without a history of MI, in contrast to patients with or without AF who had the same. However, they could not show any linear relationship between duration over six and 24 hours of AHRE and higher incidence of MACEs. That relationship between longer AHRE and MACE was found by Pastori et al. In their study, they found that patients who developed a long AHRE over 24 hours had nearly a twofold higher risk of MACE [14]. van Gelder et al., in an ASSERT (Asymptomatic Atrial Fibrillation and Stroke Evaluation in Pacemaker Patients and the Atrial Fibrillation Reduction Atrial Pacing Trial) study, found that AHREs over 24 hours were associated with an increased risk of ischemic stroke or systemic embolism and suggested that the risk of stroke was lower in patients with AHRE than those with AF [19]. Taking into consideration the aforementioned information and their data, Camm et al. suggested that patients with AHREs lasting more than 24 hours should be considered for OAC initiation [20]. The European Heart Rhythm Association (EHRA), in a consensus document in 2017, suggests that for patients presenting with AHRE and a CHA2DS2-VASc score of 0 in males and 1 in females, no OAC therapy should be considered [1]. In the most recent European Society of Cardiology (ESC) guidelines, patients with AHRE should start an OAC treatment on a case-by-case basis, considering identified stroke risk factors. For patients with longer (>24 hours) AHRE episodes and an estimated high individual risk of stroke, the initiation of OAC may be considered [2]. Our study added important information for the impact of AHREs in thromboembolic events and pointed out the need for further investigations in this field. The fact that we noticed no statistical significance when we combined the two endpoints of bleeding and thromboembolic events is important, as it adds information to the clinician that the treatment of patients with AHREs should be individualized and according to the patient’s CHA2DS2-VASc and HAS-BLED scores.

Despite the ongoing research and increasing data for AHREs, many issues have not been fully established. Recent research suggests that atrial cardiomyopathy could be a possible thrombogenic source even in the absence of AF [21]. This could explain the temporal dissociation between AF and stroke. It could also justify the occurrence of thromboembolic events in patients with atrial cardiomyopathy even without the presence of AF. AHREs could be an indication of atrial cardiomyopathy, which can explain the temporal dissociation between AHREs and stroke [18,22-24]. The ongoing clinical trial ARTESiA (Apixaban for the Reduction of Thrombo-Embolism in Patients With Device-Detected Sub-Clinical Atrial Fibrillation), along with the recent findings of the NOAH-AFNET 6 trial, may help to decide if we should consider anticoagulation treatment in patients according to evidence of atrial cardiomyopathy and without a history of AF [7,8].

This study had several limitations. First, the study protocol was designed as a single-center, nonrandomized study. Thus, we could have missed unmeasured variables associated with events and outcomes were not blinded. In addition to this, the sample size was not large enough to derive comparisons of event rates and safely extrapolate results to the general population. The absence of statistically significant results could be due to the small number of patients and the fact that the initiation of OACs was not controlled blinded or randomized. Our study design could not differentiate whether OAC treatment was the causal factor for developing an event or the result of the progression of other comorbidities. Further studies in this field are needed to clarify the issue. However, we believe it is important for clinicians to be cautious and informed about AHREs because these episodes are the forerunner of AF and other cardiovascular comorbidities.

## Conclusions

Our study demonstrated that OAC therapy in patients with AHREs was not associated with a significant difference in the risk of thromboembolic and bleeding events (although the results for the thromboembolic events were marginally statistically nonsignificant between the two groups, *P* = 0.05). In a contemporary real-world cohort of patients with AHREs, patient characteristics at baseline and AHRE duration may help to intensify patient monitoring for timely decision-making and management. Moreover, bleeding events may be a marker for a higher risk of cancer diagnosis in patients with AHREs receiving OACs. These findings highlight the importance of further research in the field to optimize patient outcomes.
